# Recurrent Gonorrhea*Written at the request of The Atlanta Medical and Surgical Journal.

**Published:** 1898-09

**Authors:** Ferd. C. Valentine

**Affiliations:** Professor of Genito-Urinary Diseases, New York School of Clinical Medicine; Genito-Urinary Surgeon, West Side German Dispensary; Genito-Urinary Consultant, United Hebrew Charities, etc., etc.


### ATLANTA
### Medical and Surgical Journal.

VOL. XV. SEPTEMBER, 1898. No. 7.

DUNBAR ROY, A.B., M.D., EDITOR.
M. B. HUTCHINS, M.D., BUSINESS MANAGER.

### ORIGINAL COMMUNICATIONS.

### "RECURRENT" GONORRHEA.*

BY FERD. C. VALENTINE, M.D.,

Professor of Genito-Urinary Diseases, New York School of Clinical Medicine; Genito-Urinary Surgeon, West Side German Dispensary; Genito-Urinary Consultant, United Hebrew Charities, etc., etc.

RÉSUMÉ.

1. Cessation of the symptoms of clap does not prove that the case is cured.

2. No female cured of the evidences of clap should be dismissed without proving that the apparently normal urethra, Bartholini's glands, the cervix and the sub-mucous tissues (especially those of the cul-de-sac) are free from gonococci.

3. No male should be dismissed from treatment until it is definitely ascertained that his urethra, seminal vesicles and prostate are free from the disease.

4. The methods of securing positive evidence of the cure of gonorrhea are within the general practitioner's reach.

5. The treatment of recurrent gonorrhea is not difficult, nor does it require special skill.

*Written at the request of THE ATLANTA MEDICAL AND SURGICAL JOURNAL.

At the onset it is well to acknowledge that the title of this paper is a misnomer, expressing, as it does, merely one manifestation of residual gonorrhea.

The general practitioner is as well acquainted with the condition as is the specialist. In many a case apparently cured of gonorrhea a discharge suddenly presents weeks, occasionally months, after the last evidence of disease was observed. This happens sometimes after coitus, sometimes without; after a glass of beer, after transient erotic excitement, or without any apparent provocative cause. Unless one knows the patient well, such a case may tax the practitioner's faith, if he would not deem the recurrence a new infection.

Oftentimes, indeed, microscopic examination shows such a suddenly appearing discharge to be loaded with gonococci, grouped in the manner as they are most frequently in a recent infection. These microbes are sometimes also disseminated throughout the discharge, or they appear scattered only.

The principal causes of such a "recurrent" gonorrhea are:

1. Marital reinfection.
2. Infarction of crypts, glands, or follicles of the anterior urethra.
3. Chronic residual posterior gonorrheal urethritis.
4. Gonorrheal prostatitis.
5. Seminal vesiculitis.

Any two or all these causes may be united in one case.

Whatever the cause, irrigations may soon bring about their apparent cure. And the disappearance of all symptoms of disease may be so rapid as to cause the patient and the physician to deceive themselves into believing that a wonderful cure has been effected. The young practitioner especially should be warned against taking even a succession of such cases into statistic consideration, unless in each case he has assured himself that the patient is free from the causes of recurrence. To briefly discuss them:

1. *Marital reinfection.* The evidences of gonorrhea in a woman may be so slight as to be imperceptible. In another paper* I endeavored to outline the methods by which the ability of a woman to

*Valentine: Residual Gonorrhea in Women. *Journal of Gynecology and Surgery*, Sept., 1898.

infect can be determined, even if she have no discharge at all, and where none can be obtained by expressing the urethra, Bartholini's glands, or scraping the cervix. The hopelessness of obtaining a cure in a man who is continually exposed to marital reinfection is too evident for discussion.

2. *Infarction of crypts, glands, and follicles of the anterior urethra.* Experience daily shows patients so far returned to apparent health that the urine is perfectly free even from granules. Months, years, may go by, without any results from provocative cause. Then suddenly the urine, from constitutional causes, becoming irritant to the urethra, reawakens its susceptibility to the gonococci that remained quiescent within its crypts, glands, and follicles, and an apparently new gonorrhea springs up. One of many cases in point may serve to illustrate this. A gentleman, aged thirty-four, acquired gonorrhea in his eighteenth year. When he was twenty-eight he married, having had no manifestation of the disease for ten years. Shortly after his marriage,* as so often happens, he had what appeared to be a very severe fresh attack of gonorrhea. His wife was similarly affected. Conscious that for six months before he had not exposed himself to infection, and his wife but recently having been a virgin, he attributed their illness to that mysterious, albeit often quoted cause, a "strain," for which he sought no treatment until violent orcho-epididymitis bound him to his bed. It had progressed to suppuration, which caused the destruction of one testicle and epididymis.

After this he had no further evidence of disease until five years later. His wife then had returned home after an absence of several weeks. Their first coitus was, within four days, followed by acute gonorrhea in both. Never having been guilty of infidelity, he suspected her, with the usual result of a family disruption. This lasted until it was shown him that either or both could harbor gonococci for years without any appreciable manifestation thereof. In both, the disease yielded rapidly to irrigations. The wife, on subsequent examination, was found to be free from the disease. The husband, however, three weeks after responding negatively to all

*Valentine: "When May Gonorrheal Patients Marry?" *American Medico Surgical Bulletin*, October 1st, 1895.

tests,* when examined urethroscopically, showed some enlarged, gaping glands. Their contents being expressed with Kollman's spatula, showed gonococci, which, with an adequately exciting cause, would have sufficed to produce an apparently fresh clap. After electrolysis † of these glands the patient resumed relations with his wife and his usual mode of high living. Examination of the entire genito-urinary apparatus six months later showed no abnormal condition, except, of course, the destroyed testicle and epididymis.

3. *Chronic residual posterior gonorrhea.* As described in another paper,‡ this cause of auto-reinfection is perhaps the most obscure, the most difficult to diagnose, but not very difficult to treat. Its precise differentiation requires, however, something beyond ordinary experience in urethroscopy, and therefore is more proper for discussion in a paper written for specialists in genito-urinary diseases.

Roughly, though, it may be said that when the first morning urine is free from even granules, expression of the posterior urethra may detach sufficient flakes to be carried in the first urine. This, centrifuged and examined microscopically, may reveal gonococci, of which no suspicion could be otherwise obtained.

The technique of expression of the posterior urethra is simple enough for even a tyro to perform. It in nowise differs from massage of the prostate and stripping the seminal vesicles (to be outlined further on), except that to obtain certainty regarding the location of the affection, pressure upon the prostate and vesicles must be avoided.

4. *Gonorrheal prostatitis.* In a brilliant paper on the subject, Wossidlo,§ of Berlin, urges that no case of gonorrhea should be dismissed without assurance being obtained that the prostate is free from disease. Perhaps the majority of cases of what I, for want

*Valentine: "The Proofs of Cure of Gonorrhea." *Clinical Recorder*, April, 1898.

†Valentine: "Chronic Gonorrhea: Its Scientific Treatment." *Clinical Recorder*, January, 1898.

‡Valentine: "A Contribution to the Study of the Symptoms of Chronic Gonorrhea." Read before the American Medical Association, Denver, June, 1898. Will soon be published in the *Journal of the American Medical Association.*

§Wossidlo: "Chronic Prostatitis." Read before the American Medical Association, Denver, June, 1898, soon to be published in the *Journal of the American Medical Association.*

of a better term, have called "recurrent" gonorrhea, are due to prostatic invasion.

It is interesting and important to note the length of time the prostate can hold gonococci without any manifestation whatever. At the risk of unduly lengthening this paper I will cite an illustrative case:

A gentleman had an attack of gonorrhea in his eighteenth year. At twenty-six he married. His wife bore him two healthy children. When he was forty-three, his wife not having become pregnant for ten years, he was taken with salpingitis at about the same time that he became affected with evidence of prostatic enlargement, such as diminution of the force of the stream, frequent nocturnal urination and inability to entirely empty his bladder. Mere examination of the enlarged gland brought forth a very small quantity of a grayish muco-pus, which was found replete with gonococci. So here is a case in which, for twenty-five years, the prostate held gonococci without any manifestation whatever, not even preventing the procreation of two healthy children.

The diagnosis of prostatitis will be outlined, together with that of seminal vesiculitis.

5. *Seminal vesiculitis*, if gonorrheal, as it is in the majority of cases, may be the cause of recurrent clap. Its other symptoms, even more so than those of prostatitis, supply that vast array of manifestations so often diagnosed as neurasthenia.

If, in a case of recurrent gonorrhea, marital reinfection can be excluded by examination of the woman; b, infarction of the crypts, glands, and follicles by urethroscopy; c, chronic residual posterior gonorrheal urethritis by expression of the posterior urethra and posterior urethroscopy; d, gonorrheal prostatitis by massage of the prostate, then we must look for gonorrheal vesiculitis.

The local manifestations of the three last mentioned conditions are grossly the same, differing only in detail.

For such an examination I deem it best to place the patient on a sofa, lying on his back. The apex of the index-finger and the bed of the nail being tightly packed with soap and then thickly anointed with vaseline, the finger is gently inserted into the rectum. The other hand rests above the pubis to steady and press down the pelvic viscera.

The pulp of the finger is turned toward the front of the patient, and lightly outlines the prostate, but exercises not even the slightest pressure upon any part of it. If the operator desires to elicit evidence of

*Chronic residual posterior gonorrhea,* he lets the finger glide from the prostate and exercises pressure, with increasing force in a stroking motion forward from the lowermost margin of the prostate, endeavoring with each stroke to force the posterior urethra against the posterior aspect of the pubis.

The patient is then ordered to urinate, and if this is the region affected, the urine will contain flakes, perhaps even filaments or shreds, which the urinary stream was unable to detach. Microscopy of these products of massage will reveal the character, probably gonorrheal, of the posterior urethritis. If this results negatively, examination for

*Prostatitis* may be made in the same manner, several days later. The size, shape, and hardness or softness of each lobe, as well as of the isthmus, should be ascertained; lobulation or smoothness should be elicited, depressible points located, and the prostatic juice, if any exudes from the meatus, microscopically examined. If none escapes, the first urine the patient passes after this massage of the prostate should be centrifuged for examination. If the prostate contains gonococci, they will be found either in the discharge that escapes from the meatus or flows into the posterior urethra or bladder and is carried off by the urine. If, however, the prostate is found to be normal, then the patient should be examined two or three days later for

*Seminal vesiculitis.* This is done in the same manner, except that the finger is passed up the rectum further, beyond the prostate and to its sides. In health the seminal vesicles can be barely, if at all, felt. When enlarged by disease, they assume the shape of more or less tensely filled little sausages. In engaging the finger as high up as possible on these bodies, by curving the finger downward and towards the center with increasing force, the seminal vesicles may be stripped of their contents. These are treated in the manner as described under the examination for prostatitis.

The first of either of these examinations is usually attended with some pain, but the relief the patient experiences is usually so great that he will ask for its repetition.

As in all other genito-urinary work, gentleness in these manipulations cannot be too strictly followed. Nothing is gained by violence, even harm can be done. Physicians unable to devote the most exquisite gentleness and sympathy to these cases would do well to relegate them to others.

The treatment of recurrent gonorrhea should be directed to its cause, or, perhaps better, its location.

Where *marital reinfection* is the cause, attempts to cure the husband must prove futile while the wife remains ill, as in the majority of instances prohibitions regarding coitus are of no avail.

Where the *crypts, glands or follicles of the anterior urethra* harbor gonococci, if systematic dilatations and irrigations do not entirely suffice, electrolysis will complete the cure.*

When posterior urethritis, prostatitis, or seminal vesiculitis causes the exacerbations of auto-reinfection, Kollman's posterior dilator, massage of the prostate, and stripping of the vesicles will be required. Irrigations, as elsewhere described, will be found valuable adjuvants to the treatment. While it is not at all likely that even the most copious irrigations will wash away enough of the *materies morbida* to materially affect the disease, they procure an artificial edema which renders the mucous membrane an unfavorable culture-medium for the gonococci.

In all cases the general condition, as well as the nervous system, suffers deterioration. This must be met by constitutional treatment, tonics, baths, attention to digestion, indeed every means at our command to fortify the patient's resistance against further inroads of the disease.

*242 West 43d Street, New York.*

*Valentine: "Chronic Gonorrhea: Its Scientific Treatment." *Clinical Recorder*, January, 1898.

